# Supplementary material for: Current limitations in predicting mRNA translation with deep learning models
Source: Genome Biol. 2024 Aug 20;25:227. doi: 10.1186/s13059-024-03369-6 (PMC11337900; doi:10.1186/s13059-024-03369-6)
Supplement: Supplementary file 1 — Additional file 1: Contains Supplementary Figures S1 - S8 as well as the description of Supplementary Table S1. For the sake of readability, we did not display all 7238 lines in the additional file. Instead, the table is provided extra as supp_tab_1.tsv. [file 13059_2024_3369_MOESM1_ESM.zip › additional_file1.pdf]

Additional File 1 to: Current limitations in predicting mRNA translation with deep learning models

Niels Schlusser[1*], Asier González[1,2], Muskan Pandey[1,3], Mihaela Zavolan[1*]

[1]Biozentrum, University of Basel, Spitalstrasse 41, Basel, 4056, Switzerland.
[2]Departament de Bioquímica i Biologia Molecular and Institut de Biotecnologia i Biomedicina, Universitat Autònoma de Barcelona, 08193 Cerdanyola del Vallès, Spain.
[3]Current address: Institute of Molecular Biology and Biophysics, Departament of Biology, ETH Zurich, 8093 Zurich, Switzerland.

*Corresponding author(s). E-mail(s): niels.schlusser@unibas.ch; mihaela.zavolan@unibas.ch;
Contributing authors: a.sevine@unibas.ch; muskan.pandey@unibas.ch;

1 Supplementary Figures

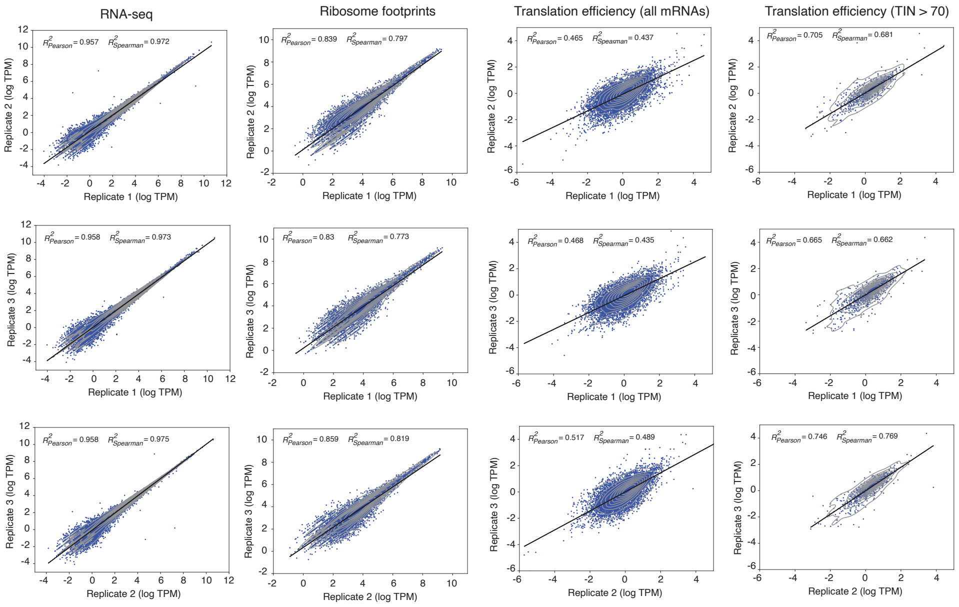

Fig. S1 Inter-replicate reproducibility of different experiments in HEK 293 cells: mRNA-sequencing coverage of the CDS (col. 1), ribosome footprint coverage of the CDS (col. 2), translation efficiency (TE) as measured by the ratio of the former two (col. 3), and TE of transcripts with TIN > 70 (col. 4).

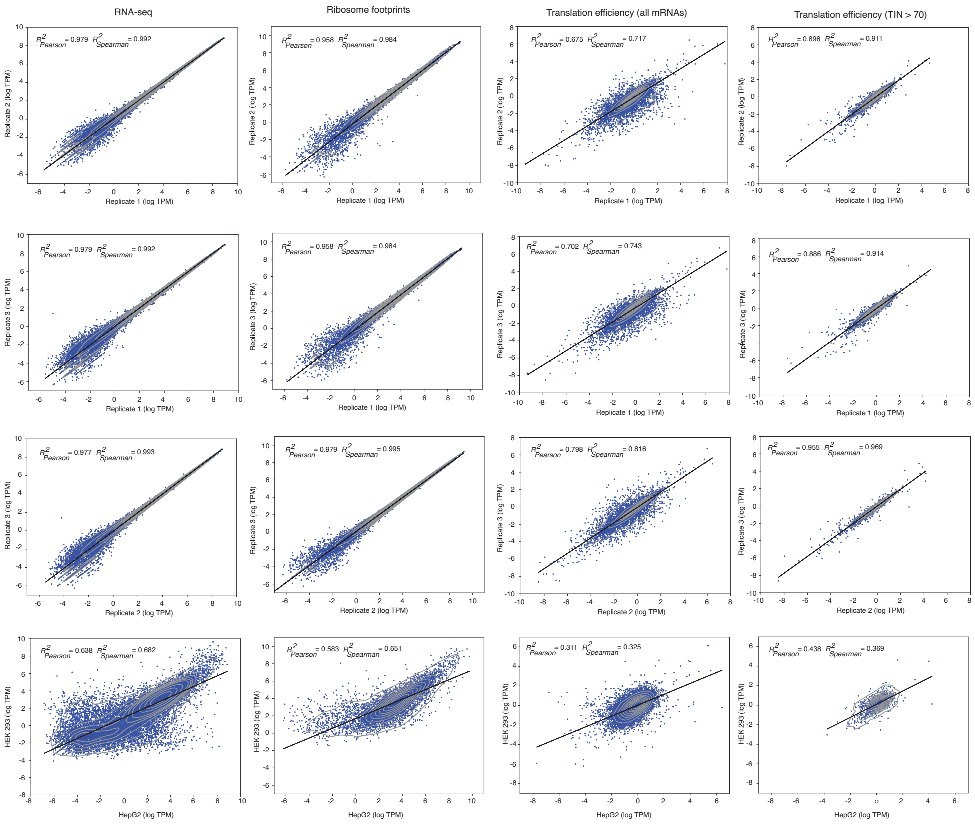

Fig. S2 Inter-replicate reproducibilty of HepG2 cell line and inter-tissue correlation between HEK 293 and HepG2 cell line: mRNA-sequencing coverage of the CDS (col. 1), ribosome footprint coverage of the CDS (col. 2), translation efficiency (TE) as measured by the ratio of the former two (col. 3), and TE of transcripts with TIN > 70 (col. 4). Correlation between replicates 1 and 2 in line 1, replicates 1 and 3 in line 2, 2 and 3 in line 3, and HepG2 and HEK293 in line 4.

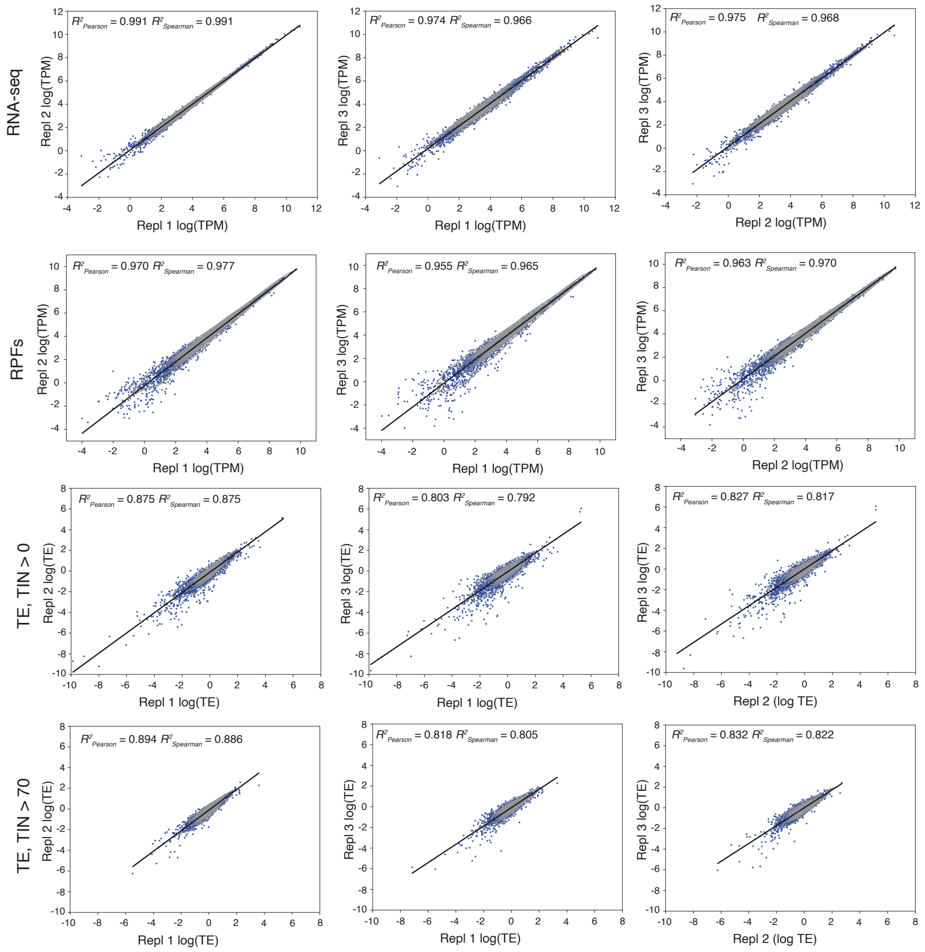

Fig. S3 Inter-replicate reproducibility of yeast: mRNA-sequencing coverage of the CDS (l. 1), ribosome footprint coverage of the CDS (l. 2), translation efficiency (TE) as measured by the ratio of the former two (l. 3), and TE of transcripts with TIN > 70 (l. 4). Correlation between replicates 1 and 2 in line 1, replicates 1 and 3 in line 2, 2 and 3 in line 3

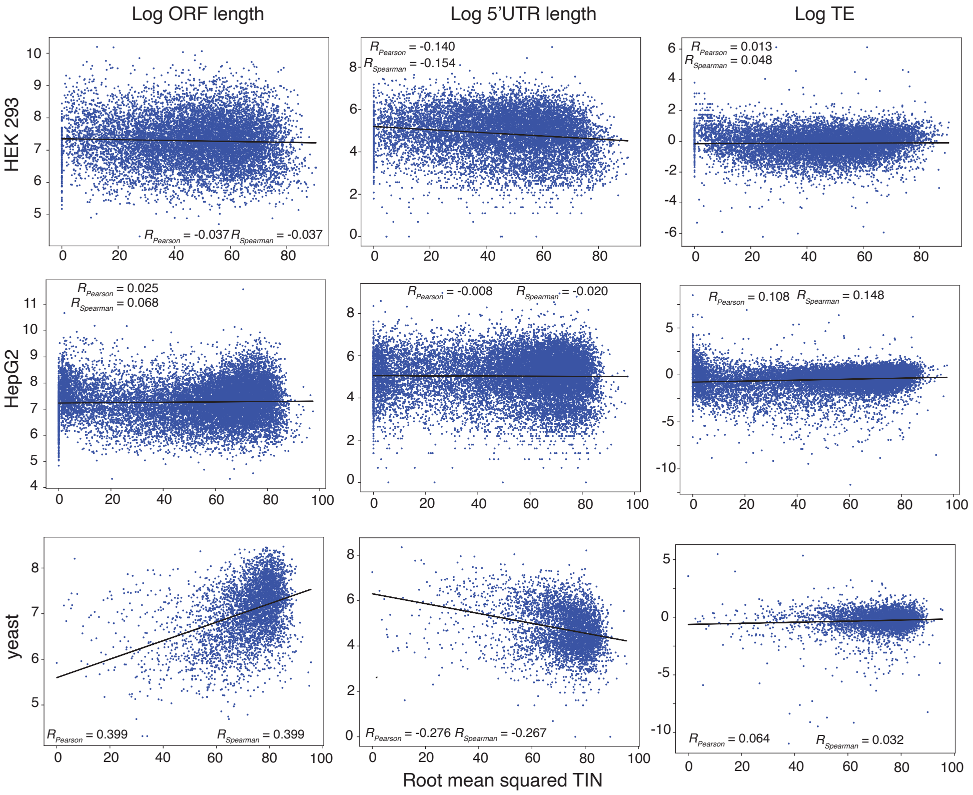

Fig. S4 Correlation of the root mean squared of the TIN with the natural logarithm of main ORF length, the 5'UTR length, and the translation efficiency.

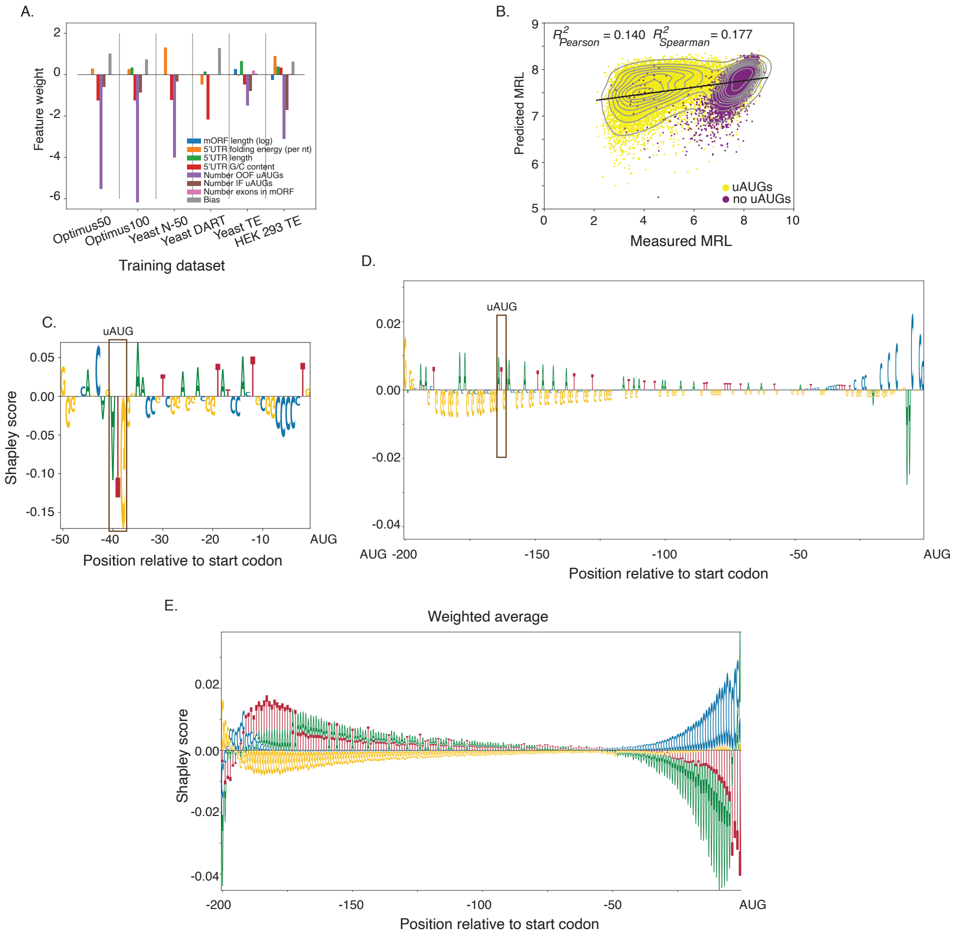

Fig. S5 Weights of the linear model associated with the different non-sequential features when being trained on different data sets (A). Upstream AUGs are by far the most influential feature. When training Optimus 5-Prime on the sequences of the optimus50 data set that do not contain uAUGs, it loses most of its predictive power (B) on a test set with both, sequences with (yellow) and without (purple) uAUGs. (C) Contributions of single nucleotides in of a test sequence containing a uAUG from the optimus50 data set, visualized using the SHAP package [1], with clear inibitory effect of uAUG on predicted MRL. (D) Evaluation of single nucleotide contributions on prediction of a uAUG-containing sequence from the HEK293 test set (high TIN): Effect of uAUG not clear. Normalized superposition of 200 test sequences longer than 200nts (E) shows position-dependent sequence bias: poly-G stretches inhibit, A/U-rich domains seem to promote translation. C-rich sequences in the vicinity of the start codon favor translation, too.

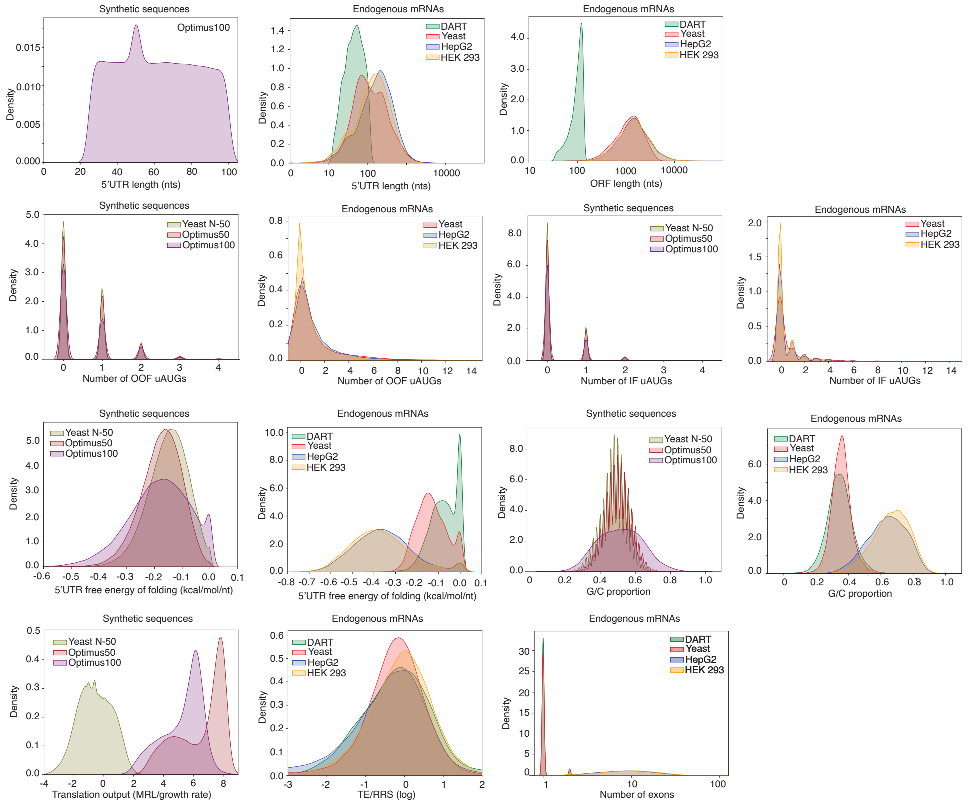

Fig. S6 Density plots of non-sequential features in the six different data sets used in this study. Synthetic data sets are Yeast50 [2], Optimus50, and Optimus100 [3]. Endogenous data sets are DART [4], endogenous yeast [5], and endogenous HEK293 [6]. Non-sequential features are the 5' UTR length (cols. 1,2, l. 1), the mORF length (col. 3, l. 1), the number of out-of-frame (OOF) uAUGs (cols. 1,2, l. 2), the number of in-frame (IF) uAUGs (cols. 3,4, l.2), the 5'UTR folding energy per unit base (col2. 1,2, l. 3), the G/C-content fraction (cols. 3,4, l. 3), and the number of exons (col. 3, l. 4). The translation output as measured by mean ribosome load (MRL) or yeast growth rate (col. 1, l. 4), and log ribosome recruitment score (RRS) or log translation efficiency (TE) (col. 2,l. 4) is also shown.

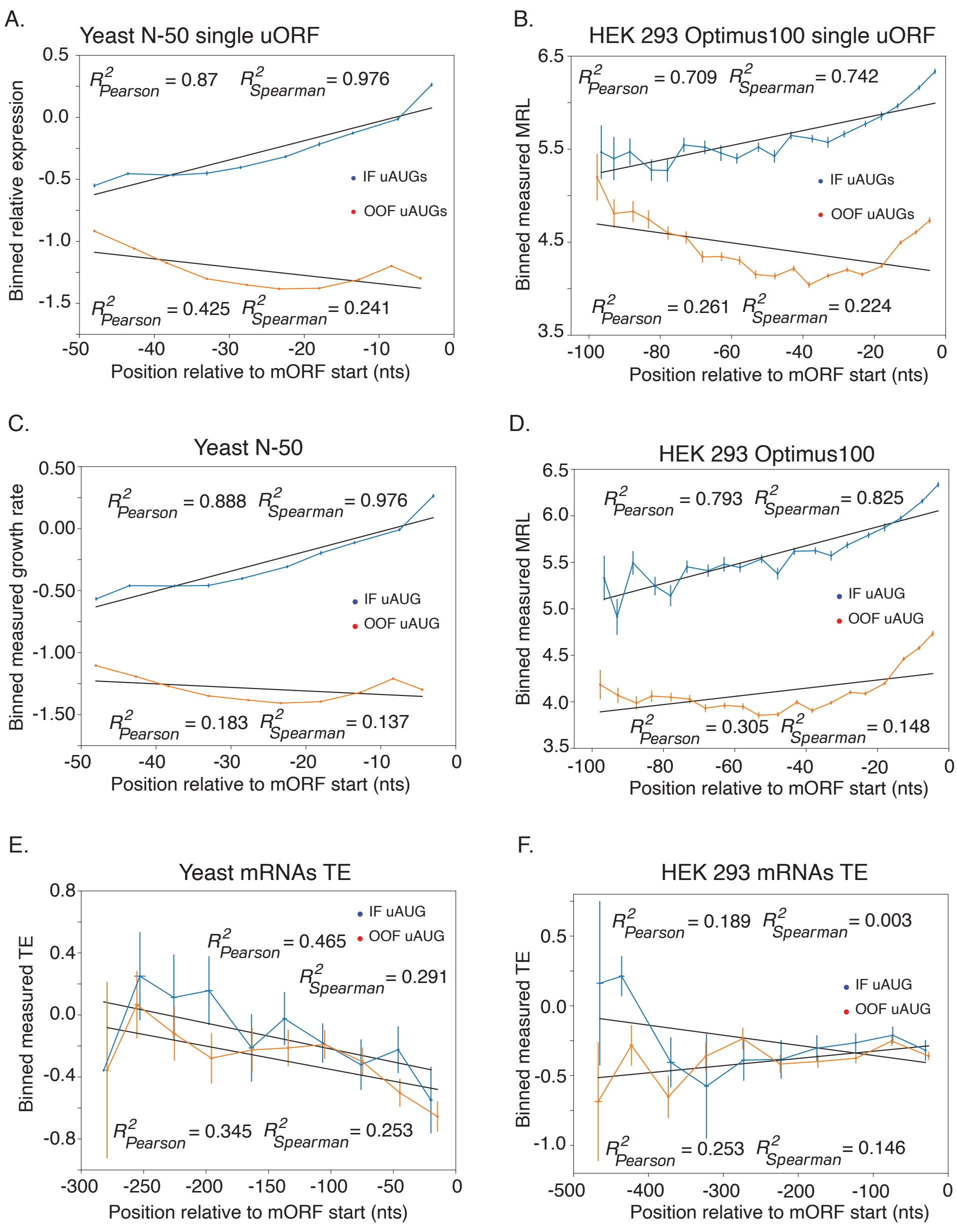

Fig. S7 The distance of IF and OOF uAUGs to the start of the mORF determines the inhibitory strength, as can be seen for yeast MPRA data (A, C), and HEK 293 MPRA data (B, D). Endogenous data yeast data are shown in (E), HEK 293 data in (F). Panels (A) and (B) display only data from transcripts with a single uAUG, whereas (C-F) use data from transcripts with at least one uAUG (either only IF or OOF). The computation of the position is based on the most upstream AUG, the measure for translational output is computed as the average over all transcripts with relative distance of the uAUG from the start codon within one of the 10 bins over the displayed range of relative separations.

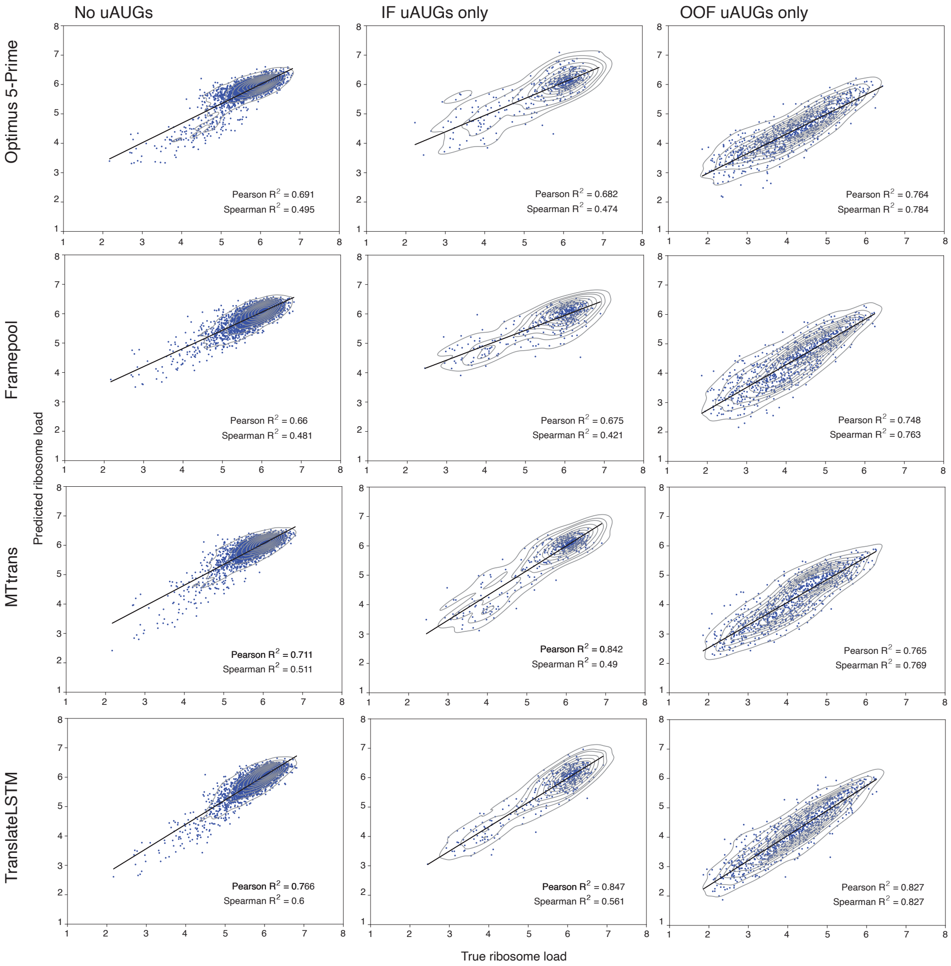

Fig. S8 The Optimus100 data set was split into three subcategories: 5'UTRs containing no uAUGs at all, 5'UTRs containing at least one IF uAUG, but no OOF uAUGs, and 5'UTRs containing at least one OOF uAUG, but no IF uAUGs. We evaluate the performance of the three existing deep learning architectures (Optimus 5-Prime, FramePool, MTtrans), as well as the new architecture, TranslateLLM, on the three data sets. TranslateLLM predicts all three subsets with the highest accuracy and leads to the most evenly shaped contour plots.

2 Supplementary Tables

Supplementary Table S1: clinvar 5'UTR variants [7], their predicted log-fold change in TE (LFC TE), clinical significance, mutated and parent UTR, gene and transcript ID. Attached to submission as `supp_tab_1.tsv`

References

[1] Lundberg, S.M., Lee, S.-I.: A unified approach to interpreting model predictions. In: Guyon, I., Luxburg, U.V., Bengio, S., Wallach, H., Fergus, R., Vishwanathan, S., Garnett, R. (eds.) Advances in Neural Information Processing Systems, vol. 30, pp. 4765–4774. Curran Associates, Inc., ??? (2017). https://proceedings.neurips.cc/paper_files/paper/2017/file/8a20a8621978632d76c43dfd28b67767-Paper.pdf

[2] Cuperus, J.T., Groves, B., Kuchina, A., Rosenberg, A.B., Jojic, N., Fields, S., Seelig, G.: Deep learning of the regulatory grammar of yeast 5' untranslated regions from 500,000 random sequences. Genome Res **27**(12), 2015–2024 (2017)

[3] Sample, P.J., Wang, B., Reid, D.W., Presnyak, V., McFadyen, I.J., Morris, D.R., Seelig, G.: Human 5' UTR design and variant effect prediction from a massively parallel translation assay. Nat Biotechnol **37**(7), 803–809 (2019)

[4] Niederer, R.O., Rojas-Duran, M.F., Zinshteyn, B., Gilbert, W.V.: Direct analysis of ribosome targeting illuminates thousand-fold regulation of translation initiation. Cell Syst **13**(3), 256–264 (2022)

[5] Weinberg, D.E., Shah, P., Eichhorn, S.W., Hussmann, J.A., Plotkin, J.B., Bartel, D.P.: Improved Ribosome-Footprint and mRNA Measurements Provide Insights into Dynamics and Regulation of Yeast Translation. Cell Rep **14**(7), 1787–1799 (2016)

[6] Alexaki, A., Kames, J., Hettiarachchi, G.K., Athey, J.C., Katneni, U.K., Hunt, R.C., Hamasaki-Katagiri, N., Holcomb, D.D., DiCuccio, M., Bar, H., Komar, A.A., Kimchi-Sarfaty, C.: Ribosome profiling of HEK293T cells overexpressing codon optimized coagulation factor IX. F1000Res **9**, 174 (2020)

[7] Landrum, M.J., Lee, J.M., Benson, M., Brown, G.R., Chao, C., Chitipiralla, S., Gu, B., Hart, J., Hoffman, D., Jang, W., Karapetyan, K., Katz, K., Liu, C., Maddipatla, Z., Malheiro, A., McDaniel, K., Ovetsky, M., Riley, G., Zhou, G., Holmes, J.B., Kattman, B.L., Maglott, D.R.: ClinVar: improving access to variant interpretations and supporting evidence. Nucleic Acids Res **46**(D1), 1062–1067 (2018)
