# Supplementary material for: Current limitations in predicting mRNA translation with deep learning models
Source: Genome Biol. 2024 Aug 20;25:227. doi: 10.1186/s13059-024-03369-6 (PMC11337900; doi:10.1186/s13059-024-03369-6)
Supplement: Supplementary file 2 — Additional file 2. Contains the review history [file 13059_2024_3369_MOESM2_ESM.docx]

**1^st^ round**

**Reviewer 1**

Schlusser et al. use three complementary massively parallel reporter assays (MPRAs) and translational efficiency (TE) data from endogenous RNAs to systematically compare different approaches for predicting mRNA translation. In brief, they compare a simple 8-parameter linear model as baseline with three published deep learning models (Optimus5`, Framepool, MTtrans). They observe that models trained on synthetic 5'UTRs do not perform well on predicting TE of endogenous mRNAs. Using cross-species predictions on same data type, they show that this is not due to different experimental readout. Additionally, the authors present two own models using long short-term memory (LSTM) networks, based either only on the 5' UTR sequence, or also including first 100 nt of the open reading frame (ORF) and non-sequential features such as out-of-frame (OOF) upstream ORFs. These models reach slightly higher accuracy than the existing models. Moving towards clinical application, the authors explore the models' performance in predicting SNP effects on TE, using first synthetic MPRA data and then ClinVar variants via transfer learning, and identify 110 variants with predicted impact on translation. Overall, this is a relevant and timely study with insights for basic research and applications in protein expression such as for mRNA vaccines. All analyses are carefully designed and well performed.

Specific points:

In order to evaluate the newly presented LSTM models, it would be helpful to get more detailed insights into what they learnt. Are the predictions build on reasonable information in the data? This may also help to understand why the models seem to transfer so badly between datasets.

Figure 3A summarises the results from the transfer of models between datasets but the plot is hard to read. Also, the authors could consider to remove Yeast TE from this plot which does not seem to work at all. The term "Reproducibility bound" not explained.


Minor points:

Because the published code could not be run, the authors newly implemented MTtrans according to descriptions, resulting in a model that is similar but not identical. Since they end up with a hugely different number of parameters compared to the original publication, it is not clear whether this obtained model can really be compared to the others.

Page 12, line 13f: "However, overall, while absolute numbers of uAUG changes are small (328 of 7'238 variants), creation/destruction of an uAUG was associated with a predicted reduction/increase of translation output." Please show the data.

Supplementary Figure 2: Gene expression can be quite different between cell types which may have strong impact on the observed correlation. Are the TE quantifications filtered for a minimum signal in RNA-seq/ribo-seq?

The authors find that information on OOF uAUG shows the most consistent effect on TE predictions. In Supplementary Figure 4, they explore the positioning OOF and IF uAUGs relative to the start codon and conclude that the repressive effect of OOF uAUGs is largely position-independent. However, it seems that OOF uAUGs show a consistent pattern which distances of more/less effect. Also, the authors claim that "Endogenous mRNAs show the same trends, though less pronounced" (Supp. Fig. 4E-H), but I cannot see this.

The naming of the model TranslateLLM seems odd, since LLM is usually used as abbreviation for large language models based on transformers, not on LSTMs.

The authors use the highest quality data for testing (based on TIN) and the rest for training (page 4, line 14-17). Does this introduce a bias into the data? Can the authors show the difference in this strategy compared to a stratified or random split?

Page 7, line 3-7: Can the authors clarify how this effects the cross-cell line training/testing?

Page 7, line 26: By which metric do the authors measure the spread around the diagonal?

Page 8, line 47: How many epochs do the authors use?

Page 11, line 32: It seems that this LSTM model is not further described and is missing in Figure 2.

Please extend the legend of Supplementary Figure 4 to clearly explain what is shown.

Supplementary Table 1 was not available for review.

Please add version numbers for all software used and avoid terms like "latest annotation" (Ensembl v105 was released in 2021).

Page 22, line 9: What learning rate was used for the adam optimiser?
Page 22, line 10: What threshold was used for the early stopping criterion?


Figures:
Please rasterise all scatter plots in the Supplementary Figures. It is currently almost impossible to open the pdf file.
Supplementary Figure 4B: Colour legend is missing.

Typos:
Supplementary Figure 4G: Formatting of correlation metrics is wrong.
Supplementary Figure 1, last line of legend: Wrong symbol.
Page 9, line 6: "generally different"
page 10, line 46: " subset of remaining constructs that did contain uAUGs"; should be "did not contain"?
Page 11, line 34: "more accurate than"
Page 16, line 20f: check sentence: "Experiments in a single cell type such as a cell line may not yield sufficiently many reliably measured 5'UTRs to train models such as TranslateLLM can be retrained."
Page 18, line 34: "UTR length as"
Page 22, line 38: "only only"

**Reviewer 2**

This manuscript by Schlusser et al. offers a comprehensive comparison of published machine learning (ML) models in predicting the translation of yeast and human transcripts from mRNA sequence alone. They provide a nice introduction and review of methods to measure and model these data. The authors make the compelling argument that although models can capture information from multiple parallel reporter assays on random sequence, they cannot then predict endogenous translation. They provide some insights into why this is the case, pointing the way forward for designing experiments to capture this information and models to learn this information. They compare models from yeast to models from human cells and find that there is significant learning that transfers, on synthetic data at least. They argue that current ML models are highly overparameterized and are learning simple rules from synthetic data that don't really capture endogenous initiation mechanisms. Overall, it draws together several different approaches, synthesizes them, and when they fail, poses the challenge of what we need to do better.

I have one significant concern:

The authors raise the important issue of how reliable and reproducible the experimental data are that we use as inputs for ML models. With regards to the translational efficiency (TE) measurements from ribosome profiling: I am concerned that the ribosome profiling data that they use from reference 19 are unusually noisy (R2 for RPFs about 0.80). I noted that the authors own RPF data from the other cell line (HepG2) were much better (R2 > 0.96). These are numbers more in line with the quality of data I expect from ribosome profiling. There are many datasets from human cell lines available publicly, including many HEK293 datasets. I recommend starting with ribosome profiling data that are more reproducible. These metrics are often reported in papers. Having better data could have affect the comparison across cell lines or the usability of TE data for training models.

Minor issues / suggestions:

One way to reduce noise in TE values would be to impose a threshold on the number of reads, where transcripts are only considered if they have enough TPM in the RNAseq, for example.

Page 4: I don't really understand the logic behind testing transcripts with the most abundant transcripts and training on all the rest. I recognize that this was what was done in previous ML models. Highly expressed transcripts have regulatory strategies that other transcripts don't have; how will the model learn this? Ribosome protein genes, for example, are the most highly expressed transcripts in most cells and they have TOP motifs that regulate their expression. If the training is done on all the other genes, the model will never learn about TOP motifs. I would think that the best strategy would be to train and test on the same distribution of data (with regards to expression) just leaving out some fraction for the test set.

I love Figure 3A, it is wonderful that the authors have directly compared all these ML models on all these different data sets. I confess however that I find it very difficult to read: the color labels are similar and the lines overlap and everything is so small. I suggest making it (much) bigger, picking either Pearson or Spearman and dropping the other (they aren't really that different), and dividing the figure up by lines 1-6, where they train and test on the same data, and lines 7-13 where they train on one dataset and test on another. And I'm not sure lines are the right thing, the connectivity doesn't really matter, maybe just big dots/shapes would work. These are all just suggestions, of course, in the hope of making the findings clearer to the reader.

The fact that TE values from ribosome profiling and RNAseq cannot be learned by ML is fascinating. Why not? Do we just not have enough data (a few thousand values instead of the hundreds of thousands in optimus50)? Or is it the biology? Why do we believe that TE values really represent initiation rates in a robust way? Can the authors comment on what evidence there is that this is true? Are they useful in some other assay, or measured by some other metric?

What about training on yeast DART and then predicting yeast TE?

There is too much going on all at once in the text on page 7, it is hard to follow. Breaking up Figure 3A into multiple panels could help.

Page 10 : "trained the Optimus5' model on the subset of remaining constructs that
did contain uAUGs." I believe they intended to write "did not contain AUGs."

**Authors’ response**

Thank you for the insightful comments and questions, which we have taken into careful consideration in revising our manuscript, as described in our point-by-point response response below.

To enable easy identification of changes relative to the first version, we highlighted them in red, where they are not editorial (correcting typos, re naming the model to translateLSTM). We provide editable figures as pdf files (referee #1 pointed out that the figures take long to open, we would like to wait with rasterizing them until the final version) together with the pdfs of the main manuscript and supplementary materials, as well as the corresponding latex and bibtex files with activated track changes. We re leased the data in the repository with Bioproject ID PRJNA1045106 to the public domain.

Referee #1:

Specific points:

In order to evaluate the newly presented LSTM models, it would be helpful to get more detailed insights into what they learnt. Are the predictions build on reasonable information in the data? This may also help to understand why the models seem to transfer so badly between datasets.

We did not find good options for decoding the information learned by LSTMs. However, to address the reviewer’s question to some extent we used the SHAP package [1] to visualize the prediction procedure of an LSTM. We applied this to both synthetic sequences from the Optimus50 and endoge nous 5’UTRs measured in HEK 293 cells (see Suppl. Fig. 6C-E). As the package requires that sequences have fixed length, for the endogenous se quences we used the 200 nucleotides upstream of the start codon in 5’UTRs that were at least 200 nucleotides long. The package was able to reveal the inhibitory effect of uAUGs in the Optimus50 dataset, but not in the endogenous 5’UTRs, as shown by the difference in estimated impact of the 3 nucleotides in the AUG sequence (Suppl. Fig. 6). To better understand what features of the endogenous sequences the LSTM learns, we carried out the analysis of 200 5’UTR segments (from the mRNAs with the highest TIN score) and summarized the SHAP scores for each nucleotide and each position along these sequences. The summary (also shown in Suppl. Fig. 6) suggests that C nucleotides immediately upstream of the AUG have a positive impact on translation, whereas G nucleotides located distally from the AUG, at the 5’ end of the 5’UTRs have a repressive effect on translation. Data from prior analyses do indicate that a C-rich element immediately up stream of the start codon promotes translation, but more work is needed to understand the mechanisms behind these predicted impact of elements identified by the SHAP algorithm.

Figure 3A summarises the results from the transfer of models between datasets but the plot is hard to read. Also, the authors could consider to remove Yeast TE from this plot which does not seem to work at all. The term ”Reproducibility bound” not explained.

Fig. 3a: We better explained the reproducibility bound in the figure caption and replaced the yeast data set with a more extensive and reproducible one. We would like to keep the yeast data for completeness.

Minor points:

Because the published code could not be run, the authors newly implemented MTtrans according to descriptions, resulting in a model that is similar but not identical. Since they end up with a hugely different number of pa rameters compared to the original publication, it is not clear whether this obtained model can really be compared to the others.

We share the referee’s concern about the comparability of our implementa tion of MTtrans with the original. However, while the code in the repository did not run out of the box, we made a big effort implementing their archi tecture in the keras/tensorflow framework to ensure comparability with the other architectures from the code and documentation in their github reposi tory [2]. We further checked that the performance of our implementation of MTtrans is in line with what the authors reported for their implementation on synthetic datasets. Therefore, we decided to rather keep our implemen tation of MTtrans rather than not considering this tool at all.

Page 12, line 13f: ”However, overall, while absolute numbers of uAUG changes are small (328 of 7’238 variants), creation/destruction of an uAUG was associated with a predicted reduction/increase of translation output.” Please show the data.

We modified Fig. 4C such that the number of variants creating or breaking uAUGs in each bin are displayed in the histogram.

Supplementary Figure 2: Gene expression can be quite different between cell types which may have strong impact on the observed correlation. Are the TE quantifications filtered for a minimum signal in RNA-seq/ribo-seq?

 We did not apply such a filter. The replicates were generally well correlated and we aimed to obtain a dataset as comprehensive and unbiased as possible.

The authors find that information on OOF uAUG shows the most consistent effect on TE predictions. In Supplementary Figure 4, they explore the po sitioning OOF and IF uAUGs relative to the start codon and conclude that the repressive effect of OOF uAUGs is largely position-independent. How ever, it seems that OOF uAUGs show a consistent pattern which distances of more/less effect. Also, the authors claim that ”Endogenous mRNAs show the same trends, though less pronounced” (Supp. Fig. 4E-H), but I cannot see this.

We rephrased the paragraph about the position dependence of out-of-frame (OOF) and in-frame (IF) upstream AUGs (uAUGs) more accurately.

The naming of the model TranslateLLM seems odd, since LLM is usually used as abbreviation for large language models based on transformers, not on LSTMs.

Indeed, LLMs are usually transformer-based architectures. Although LSTMs were previously also quite popular in natural language processing (NLP), we agree with the referee that the name TranslateLLM might be misleading. Therefore, we renamed the new, composite architecture TranslateLSTM.

The authors use the highest quality data for testing (based on TIN) and the rest for training (page 4, line 14-17). Does this introduce a bias into the data? Can the authors show the difference in this strategy compared to a stratified or random split?

Previous models like Optimus 5-prime have been using the coverage as split ting criterion, presumably to ensure that the performance estimate inaccu racies come mostly from the model, not the test data. To stay in line with this philosophy we also sought to select the best quality data for testing. It is not entirely clear how to select the highest quality TE data. Since selection based on coverage in ribo-seq introduces a bias towards large TEs while selection by TIN in RNA-seq does not, we decided to use the TIN score as splitting criterion. Nevertheless, we do share the referee’s concern with this strategy in general and for this reason we verified that the TIN score does not introduce a bias in HEK293 cells. We also checked random splits and stratified splits in TIN and TE, and found that neither signifi cantly change the result as elaborated in the text. For yeast, where there is indeed a bias towards higher TEs, shorter UTRs and longer main ORFs in the higher TIN transcripts, we used three different random splits to evaluate the performance.

Page 7, line 3-7: Can the authors clarify how this effects the cross-cell training/testing?

There is no effect on cross-cell-line applications, since cross-cell-line experi ments are performed with the yeast50 and optimus50 data sets, both having fixed length and therefore same number of input parameters. We added a sentence to the mentioned paragraph to clarify.

Page 7, line 26: By which metric do the authors measure the spread around the diagonal?

We used the sum of the differences between predicted and measured MRL over test transcripts, which we also report in the text.

Page 8, line 47: How many epochs do the authors use?

We added the training specifications to the text.

Page 11, line 32: It seems that this LSTM model is not further described and is missing in Figure 2.

Since this model is very similar to the 5’UTR column of Fig. 2D, we did not draw it explicitly in the initial version of the figure. We have now added a panel with the LSTM layout to Fig. 2.

Please extend the legend of Supplementary Figure 4 to clearly explain what is shown.

We expanded the caption and explained the figure content better.

Supplementary Table 1 was not available for review.

We are not sure why the table was not there, we upload Supp. Tab. 1 together with the resubmission.

Please add version numbers for all software used and avoid terms like ”latest annotation” (Ensembl v105 was released in 2021).

In the revised manuscript we supply the version numbers for used software and avoid relative terms like ”latest annotation”.

Page 22, line 9: What learning rate was used for the adam optimiser? The learning rate of the Adam optimizer was 0.001, the default value, which we found to yield superior performance relative to other values or more complex learning rate schedules. We added the value to the corresponding paragraph in the Methods section.

Page 22, line 10: What threshold was used for the early stopping criterion? We use a callback instance from the keras-tensorflow framework with a pa tience of 10 epochs, which restores the optimal settings within these 10 epochs after prematurely stopping the learning process.

Figures

1. To keep the figures in an editable format we stick with pdf format. We can provide bit formats of the final versions of the figures.

2. We fixed the color legend of Supp. Fig. 4B

Typos

We thank the referee for pointing out the typos, which we have fixed.

Referee # 2:

Significant concern:

The authors raise the important issue of how reliable and reproducible the experimental data are that we use as inputs for ML models. With regards to the translational efficiency (TE) measurements from ribosome profiling: I am concerned that the ribosome profiling data that they use from reference 19 are unusually noisy (R2 for RPFs about 0.80). I noted that the authors own RPF data from the other cell line (HepG2) were much better (R2 ¿ 0.96). These are numbers more in line with the quality of data I expect from ribosome profiling. There are many datasets from human cell lines available publicly, including many HEK293 datasets. I recommend starting with ribosome profiling data that are more reproducible. These metrics are often reported in papers. Having better data could have affect the comparison across cell lines or the usability of TE data for training models.

We share the referee’s concerns about the limited reproducibility of the ribosome profiling data from [3]. We also agree with the referee that HEK293 is a well-studied cell line of which a good amount of data is publicly available. However, this dataset was the best we found to satisfy our requirements, namely

• Recent publishing date to ensure state-of-the-art sequencing depth

• Experiment performed in triplicate to allow assessment of reproducibil ity

• Matched RNA sequencing and ribosome profiling from the same sam ples to mitigate batch effects.

5

The used data set was the highest quality publicly available data set we found. We also agree the HepG2 data set within this publication is more reproducible than the used HEK293 data, but we do not have access to HEK293 data fulfilling the above mentioned criteria.

Minor issues/suggestions:

One way to reduce noise in TE values would be to impose a threshold on the number of reads, where transcripts are only considered if they have enough TPM in the RNAseq, for example.

We agree with the referee that imposing an expression threshold decreases the noise. In fact, we experimented with thresholds on expression in both RNAseq and ribosome profiling and did not see a performance increase, i.e. the positive effect of the decrease in noise is outweighed by the negative effect of having less training data. Therefore, we did not pursue this strategy further.

Page 4: I don’t really understand the logic behind testing transcripts with the most abundant transcripts and training on all the rest. I recognize that this was what was done in previous ML models. Highly expressed transcripts have regulatory strategies that other transcripts don’t have; how will the model learn this? Ribosome protein genes, for example, are the most highly expressed transcripts in most cells and they have TOP motifs that regulate their expression. If the training is done on all the other genes, the model will never learn about TOP motifs. I would think that the best strategy would be to train and test on the same distribution of data (with regards to expression) just leaving out some fraction for the test set.

As alluded to in the manuscript we share the referee’s concern about sep arating test and training data set based on expression levels, since we too believe that introduces a strong bias. Following the referee’s suggestion, tested three different random splits and stratified splits for TIN and TE in the HEK293 data (see Supplemental Figure 4). This seem to only have a minor effect on the outcome.

I love Figure 3A, it is wonderful that the authors have directly compared all these ML models on all these different data sets. I confess however that I find it very difficult to read: the color labels are similar and the lines overlap and everything is so small. I suggest making it (much) bigger, picking either Pearson or Spearman and dropping the other (they aren’t really that different), and dividing the figure up by lines 1-6, where they train and test on the same data, and lines 7-13 where they train on one dataset and test on another. And I’m not sure lines are the right thing, the connectivity doesn’t really matter, maybe just big dots/shapes would work. These are all just suggestions, of course, in the hope of making the findings clearer to the reader.

Fig. 3A: We thank the referee for the useful suggestions to make Fig. 3 more readable. Following the suggestions:

• We kept only *R*^2^_Pearson_ as a performance criterion.

• We divided the plot into two: l. 1-6 for the single data set applications, and l. 7-13 for mixed data set applications.

• We kept the lines connecting the points. While we agree that the con nectivity does not have a meaning in terms of result, we found that the lines help the reader in distinguishing different models’ performance from each other, as the individual data points are often close to each other.

The fact that TE values from ribosome profiling and RNAseq cannot be learned by ML is fascinating. Why not? Do we just not have enough data (a few thousand values instead of the hundreds of thousands in optimus50)? Or is it the biology? Why do we believe that TE values really represent initiation rates in a robust way? Can the authors comment on what evidence there is that this is true? Are they useful in some other assay, or measured by some other metric?

The reviewer’s question touches the core message of our publication. Our answer, as also reflected in a slightly modified version of the discussion, is:

• We conjecture the learning task overall does not work well because of the low abundance of representative data (either *∼* 10*^′^*000 samples of representative endogenous data or *∼* 100*^′^*000 samples of not represen tative data).

• As the referee pointed out, translation efficiency indeed is not to be equated with translation initiation efficiency. However, [4] suggest a close relationship for the majority of transcripts, the variation in translation elongation rate being much smaller than the variation in initiation rate [5]. We experimented with quantities that can be related more directly to initiation, like the relative coverage in the vicinity of the start codon. Given our data, we found that the coverage of a smaller part of the transcript is so sparse that the gain in precision is outweighed by the loss in sample size. Moreover, translation efficiency is an established concept with a simple interpretation. Therefore, we finally decided to make this approximation.

• An alternative readout and measure could be the ribosome recruitment score (RRS), as obtained by DART [6]. The data seems to be of quite high quality, as the high performance compared to similar size data sets indicates. RRS obtained by DART from human cells could be a promising future learning target.

What about training on yeast DART and then predicting yeast TE? In fact, we experimented with this strategy before and found very low perfor mance comparable to the performance of training the models on endogenous yeast data only. We therefore did not include these results. It is importantly to point out that the sequences used in the DART experiment do not have upstream AUGs, which are by far the most predictive regulatory feature in synthetic sequences (see Supp. Fig. 4(a)). The overall size of the endoge nous yeast data set seems too small to ”teach” the model about the effect of uAUGs when the model is pre-trained on DART data.

There is too much going on all at once in the text on page 7, it is hard to follow. Breaking up Figure 3A into multiple panels could help. See above

Page 10 : ”trained the Optimus5’ model on the subset of remaining con structs that did contain uAUGs.” I believe they intended to write ”did not contain AUGs.”

We corrected the error on page 10, it should indeed read ”did not contain uAUGs”.

**2^nd^ round**

**Reviewer 1**

The authors addressed all open questions.

**Reviewer 2**

The authors have thoughtfully responded to comments from the first round of review and the manuscript is improved. I have no further comments.
